# Lessons learned from participatory research to enhance client participation in long-term care research: a multiple case study

**DOI:** 10.1186/s40900-020-00187-5

**Published:** 2020-06-01

**Authors:** Aukelien Scheffelaar, Nanne Bos, Marjan de Jong, Mattanja Triemstra, Sandra van Dulmen, Katrien Luijkx

**Affiliations:** 1 Nivel (Netherlands Institute for Health Services Research), PO Box 1568, 3500 BN Utrecht, The Netherlands; 2 Department of Primary and Community Care, Radboud University Medical Center, Radboud Institute for Health Sciences, Nijmegen, The Netherlands; 3 Independent co-researcher, Amsterdam, The Netherlands; 4 Faculty of Health and Social Sciences, University of South-Eastern Norway, Drammen, Norway; 5 Tranzo Academic Centre for Transformation in Care and Welfare, Tilburg University, Tilburg, The Netherlands

**Keywords:** Participatory research, Long-term care, Co-researchers, Case study, Mental health care, Elderly care, Intellectual disability care

## Abstract

**Background:**

Although participatory research is known to have advantages, it is unclear *how* participatory research can best be performed. This study aims to report on lessons learned in collaboration with service users involved as co-researchers in three participatory teams in long-term care.

**Methods:**

A multiple case study design was chosen to explore the collaboration in three teams, each covering one specific client group receiving long-term care: physically or mentally frail elderly people, people with mental health problems or people with intellectual disabilities.

**Results:**

A good working environment and a good collaboration were found to be crucial requirements for participatory research. A good working environment was developed by discussing reasons for engagement and wishes, formulating basic rules, organizing training sessions, offering financial appreciation, and the availability of the researcher to give travel support. The actual collaboration was established by developing a bond and equal positioning, deciding on the role division, holding on to transparency and a clear structure, and have sufficient time for the collaboration. Moreover, the motivations and unique contributions of the co-researchers and differences between the teams were reported. The motivations of co-researchers ranged from individual goals – such as personal development, creating a new social identity and belonging to a social group – to more external goals, such as being valuable for other service users and increasing the quality of care. An inclusive collaboration required valuing the individual contributions of co-researchers and adjustment to team differences.

**Conclusions:**

The results showed the importance of developing a good working environment and establishing a good collaboration for participatory research. Furthermore, the study shows that individual and team differences should be taken into account. These results can be used by researchers for designing and shaping future research projects in long-term care in collaboration with co-researchers.

## Plain English summary

Although including service users actively as co-researchers in research is known to have advantages, it is unclear *how* so-called participatory research can best be performed. This study aims to report on lessons learned in collaboration with service users involved as co-researchers in three participatory teams in long-term care. Developing a good working environment and achieving a good collaboration were essential for a meaningful collaboration. A good working environment was developed by discussing reasons for engagement and wishes, formulating ground rules, organizing training, offering financial appreciation, and providing support. The actual collaboration was established by developing a bond and equal positioning, holding on to transparency and a clear structure, and have sufficient time for the collaboration. Moreover, the motivations of co-researchers ranged from individual goals – such as personal development, creating a new social identity and belonging to a social group – to more external goals, such as being valuable for other service users and increasing the quality of care. There appeared a great diversity between the individual contributions of co-researchers and differences between the teams, which shows the importance of deciding on the task divisions to take into account the various qualities of co-researchers with different strengths.

The results showed the importance of developing a good working environment and establishing a good collaboration for participatory research. Furthermore, the study shows that individual and team differences should be taken into account. These results can be used by researchers for designing and shaping future research projects in long-term care in collaboration with co-researchers.

## Introduction

In recent years, service users receiving long-term care have become more actively involved in research and quality improvement through adopting a role as co-researchers. Consequently, participatory research is being conducted ‘with’ or ‘by’ service users as co-researchers, rather than ‘to’, ‘about’ or ‘for’ as is usual [1]. This development is being driven by three core arguments. Firstly, putting the client perspective at the centre by actively involving service users in several research stages is expected to increase the quality of studies, as the research better reflects their ideas, needs and priorities and as such enhances the (content) validity and relevance [2–5]. Secondly, the active role of co-researchers yields to more effective quality improvements and enhances the support for the findings and proposed changes by clients and professionals [2, 3]. Thirdly, the ontological and normative argument concerns the rights of clients as care users and humans to influence decisions regarding their lives and specifically their care [2, 4, 6]. In this argument, service users have the democratic right to become involved [7, 8]. This is especially relevant for recipients of long-term care, who depend on the provided care for a long time. For these persons, participation in research may be an opportunity to construct a positive social identity and build up their self-esteem [7, 9].

There is a growing amount of literature about client participation. Participation in research can take many different forms, as service users can perform various tasks depending on their roles and levels of participation. Some service users are advisors in steering groups [5, 10] or involved in agenda setting [11, 12]. Others perform research activities themselves as co-researchers [13]. They can be involved in preparatory activities in research, such as formulating research topics and recruiting participants [7, 14]. Co-researchers can also be involved in data collection; they can observe and record what strikes them, or conduct interviews or focus group discussions [1, 5, 14–17]. Furthermore, co-researchers have been involved in analysing the outcomes [14, 15, 18]. In order to become experienced and well prepared, co-researchers may attend formal training, learn while performing the research, or a mixture of the two [19].

Arnstein’s (1969) ladder of participation is probably the best-known conceptualisation of variations in participation ‘levels’. The ladder distinguishes five levels of participation, ranging from informing to citizen control [20]. Some academics criticised the oversimplification of reality and the underlying assumption that higher steps on the participation ladder are deemed better while this is not always the case [2]. More recently, Bigby et al. (2014) distinguished three main roles for co-researchers, which overlap with the levels on the ladder. The *advisory role* is the commonest form of inclusion and corresponds to the consultation level. Co-researchers give advice about research priorities, the design and data collection methods in an advisory group and exert little control over how their input is used [21]. A *leading and controlling role* suggests that co-researchers initiate, lead and carry out research on their own terms (examples are [22] and [17]). A third way of involving co-researchers in research is in *collaboration or partnership* with researchers, with the initiation and leading roles not necessarily held solely by co-researchers. The position of the co-researchers is then not privileged or subordinate but equal [14, 15]. Important conditions for collaboration are time, trusting relationships, money and commitment from several parties [21].

Existing literature addresses the required competencies of researchers and co-researchers, and conditions and potential barriers for participatory research [7, 8, 23–26]. Biomedical researchers often believe that partnership in research is complex, time-consuming, and incomprehensible with the objectiveness principle [14]. Moreover, researchers judge experiential knowledge often as inferior, they tend to invite the most accessible service users to join the research, and travel support and financial compensations are not always arranged [7]. When the required circumstances and individual needs and capacities are not taken into account, well-intended attempts can result in pseudo-participation and tokenism [23]. The study of Brown et al. provided some examples of practical difficulties, as researchers tended to share an overload of written information which was not always needed, and small talk was viewed as a waste of time by researchers although it was viewed as essential for developing a bond [27]. There is still a lack of practical knowledge about *how* participatory research can best be designed and performed [28]. What are the requirements for participatory research, and how can they be applied in practice? To avoid tokenism and bad practices in attempts to achieve participatory research, a better picture of these ‘how’ issues is needed [15, 21, 28, 29]. This can be most effectively constructed from experience of what actually happens in research that aims to be participatory [15].

We recently conducted three participatory research projects in long-term care which are used for exploring the collaboration process and the motivations of co-researchers to participate [30]. The aim of this article is to report on lessons learned about collaboration between researchers and co-researchers that can be derived from the three projects. Each project involved a research team of researchers and co-researchers. The co-researchers of one team concerned physically or mentally frail elderly people, the second involved people with mental health problems and the third involved people with intellectual disabilities. This article provides insight into team members’ experiences and the requirements for collaboration in a team of researchers and co-researchers in long-term care research that focused on the quality of care relationships between service users and care professionals. The three distinct research teams from different client groups make it also possible to explore differences and similarities in client participation opportunities in three long-term care settings.

There is no consensus yet in the literature about terminology in research with co-researchers who are experts thanks to their experience. A variety of terms such as ‘inclusive research’, ‘emancipatory research‘, ‘participatory action research’, ‘patient and public involvement’ and ‘patient participation’ are used. Such terms are used interchangeably, although some specific connotations and meanings exist [31]. In this article, the term ‘participatory research’ is used to describe the process of collaboration between researchers and co-researchers in three research teams focused on quality improvement. The definition of Frankena et al. (2015) was followed: “participatory research strives for a partnership between patients and researchers, meaning that control is shared between both parties” [7]. The term “co-researchers” is chosen for those service users actively involved in the research, to emphasize the joint collaborative research process.

## Methods

### Setting the scene: the broader context

This article presents three projects performed by three teams including co-researchers and researchers in long-term care in the Netherlands. A three-year study from 2016 to 2019 was conducted to find and optimise the most suitable and useful qualitative instruments for monitoring care relationships in long-term care by co-researchers from a client perspective, as these relationships are a major determinant of the perceived quality of care. The instruments focused on care relationships between service users and care professionals who see service users most often to provide assistance, supporting care and physical care, e.g. various types of nurses, care aides and personal carers (see for study protocol [30]). A Delphi method was used to select five qualitative instruments by co-researchers and stakeholders such as representatives of care providers and branch organisations, nationwide client (council) organisations, staff from the care organisations involved, and health insurers. The content of the instruments was adapted for the research context with input from the co-researchers by adding questions about the quality of a care relationship, based on the findings of a systematic review and a qualitative research [32, 33]. The guidelines of the instruments were also adapted to give co-researchers a central participatory role in using the instruments. Co-researchers were trained to conduct interviews or focus group discussions with service users.

The central focus of this study was to find out which qualitative instrument applied by co-researchers was most useful for evaluating the quality of care relationships between service users and care professionals in long-term care by interviewing the service users. The three research teams applied and evaluated all five qualitative instruments: Am I Satisfied, Client about quality, Feedback consultation, WIEK and Participatory narrative inquiry. Co-researchers implemented the instruments, independently or with assistance of a supporting interviewer. Each of the qualitative instruments has its own unique properties and unique qualitative approach. One qualitative instrument concerns focus groups with service users, professionals and the manager of a ward, followed by follow up meetings after one month. Two qualitative instruments concern individual interviews with service users. Two qualitative instruments combine multiple methods, including individual interviews and a focus group. Three instruments provide improvement information for individual care relationships, while the results of other instruments can be used for improvement opportunities at a more aggregated group level (i.e. team, ward or organisation level). The specific content and findings of the five qualitative instruments and the performed process evaluation are reported elsewhere [34]).

### Case study design

For the design of this study, a multiple case study was chosen to explore the co-researchers’ and researchers’ role in this participatory research in long-term care. Case study research involves a ‘how’ or ‘why’ question about a contemporary set of events over which a researcher has little or no control [35]. It is an empirical inquiry that investigates a contemporary phenomenon (the ‘case’) in depth and within its context. This is especially useful when the boundaries between phenomenon and context may not be evident [35]. The essence of a case study is that it tries to illuminate a decision or set of decisions: why they were taken, how they were implemented, and with what result [35, 36]. Therefore, the orientation of the study was towards pragmatism.

The cases in this study are defined as the participation and collaboration of three teams of co-researchers and researchers in three long-term care settings. Each team consisted of two researchers and five or six co-researchers. The first team consisted of physically or mentally frail older adults (the OA team), the second of people with mental health problems (the MH team) and the third of people with an intellectual disability (the ID team). The basic characteristics and minimum capabilities needed for inclusion in a research team are described in Table 1. The reason for drawing up this profile was the intended active role in the performance of the qualitative instruments and within the research as a whole. The capabilities were also based on earlier participatory studies that came to the fore in a scoping review [8, 24, 37–40].

**Table 1:** Inclusion criteria for co-researchers

- 18 or older (no upper limit)	
- Experience as a client of long-term elderly care (residential or home care), mental health care, or care for intellectual disabilities	
- Receiving care for at least three months	
- Able to communicate verbally in Dutch	
- Able to generalise from their own experiences	
- Able to hold a conversation	
- Able to read and write at a basic level	
- A fairly stable health situation	
- Able to travel short distances	

### Research process

**Fig. 1:**
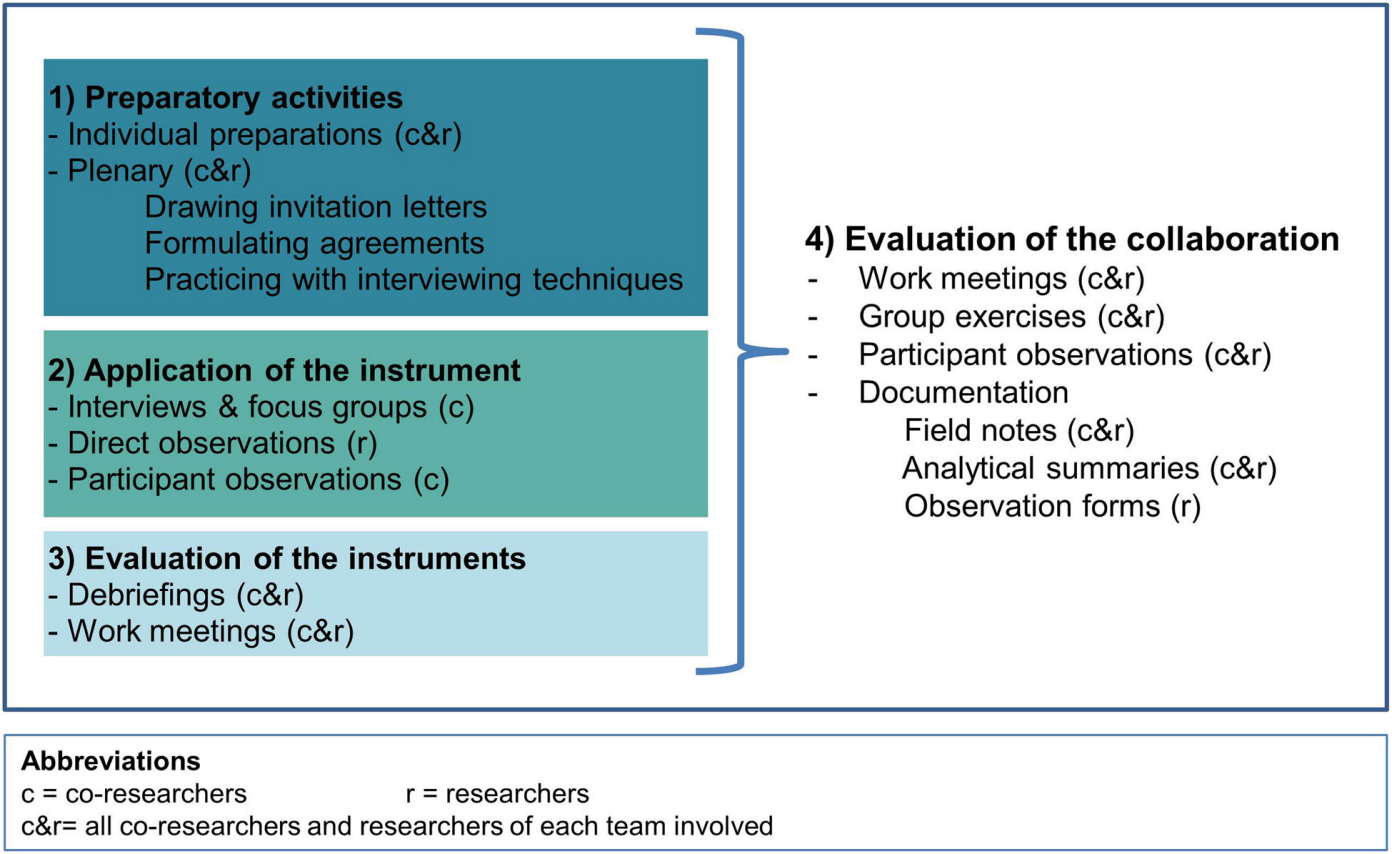
Phases of research process and data collection

The joint research process consisted of three phases: 1) preparatory activities, 2) application of the instruments, and 3) evaluation of the instruments (see Fig. 1). The preparatory activities involved drawing up the invitation letter for respondents, formulating ground rules, and training sessions. The use of the instruments by co-researchers consisted of individual interviews, group interviews, the corresponding preparation meetings and debriefings, and work meetings. The evaluation of the instruments and collaboration was carried out in work meetings by the research team.

To recruit co-researchers, invitations were sent via the contacts in the care organisations to care professionals, client councils and activity supervisors at the learning centres. Service users who were willing to join the research team as a co-researcher contacted the first author, either in person or through a care professional. More information was provided when requested, and individual introductory meetings with the first author were scheduled with those showing interest.

### Data collection

This case study draws upon the experiences of researchers and co-researchers collaborating in the three teams. Four sources of data were collected: direct observations, participant observations, individual and group discussions, and documentation. Two types of observations were made during the research process. Firstly, researchers carried out direct observations while interviews and focus group sessions were being held in order to assess the qualitative instruments. Secondly, participant observations were made in the training sessions and work meetings of the three research teams. The difference between direct observations and participant observation is associated with the researchers’ role: *passive* in the direct observations of the measurement moments, and *active* (as a research team member) in the training sessions and work meetings. Field notes were made of the salient features of both types of observations. The third source of data is the evaluation of individual and group discussions. Individual conversations between the involved co-researcher and researcher were held before and after the instruments were applied by co-researchers in practice, and concerned in particular preparations and debriefings. Three group discussions were held in each research team by its members (5–6 co-researchers and 2 researchers) about the experiences and to evaluate the participation and collaboration of co-researchers and researchers. Plenary discussions were audiotaped and field notes were made. Fourthly, various outputs from the research teams were part of the documentation, such as notes made by co-researchers, analytical summaries of the work meetings and observation forms filled in by the researchers. Some co-researchers also wrote down their experiences in notebooks. Altogether, these four sources provided a variety of data for overall reflections, to create insights into the collaboration process.

### Data analysis

Cross-case syntheses and four types of data analysis were used: categorical aggregation, direct interpretation, looking for patterns, and naturalistic generalisation [41]. In categorical aggregation, the researcher looks for a collection of instances from the data to find relevant meanings. In direct interpretation, single instances are looked at. Looking for patterns between cases means that the researcher looks for similarities and differences among cases. Lastly, naturalistic generalisations can be developed [41]. Yin (2014) described looking for patterns more thoroughly as “cross-case synthesis”. Cross-case syntheses can be performed when two or more cases are studied. Word tables can be created to display the data from the individual cases according to one or more uniform categories [35].

Direct interpretation and looking for patterns were carried out by each research team in work meetings and evaluation meetings. Each team discussed research experiences, focusing on motivations, facilitators and barriers of the collaboration. Co-researchers shared their own experiences of their roles and participation and interpretations in the research teams, and also made notes of their experiences individually. Furthermore, notes were made by the researcher of the topics discussed by the research team. The other two forms of data analysis (categorical aggregation and naturalistic generalisation) were carried out by the first author (AS), who read the raw material collected and identified similarities and cross-case patterns. The results were then discussed with other authors (NB, MdJ, MT, SvD, KL).

With regard to the data analysis, the quality of the qualitative data was validated and increased in three ways. First, the work meetings and debriefings of co-researchers and researchers in each research team enabled the inclusion of both co-researcher and researcher perspective in the analysis, which was likely to increase the internal coherence and validity of the findings. Second, one co-researcher contributed as a co-author to the writing stage of this article to ensure that the article reflected the perspectives of co-researchers well throughout. Third, the dilemma’s and questions rising during data collection and analysis were discussed in peer debriefings with all co-authors, of which some had more distance to the practical execution of the research.

### Ethical considerations

The study was submitted to the Medical Ethics Committee of the Radboud Uuniversity medical center to decide whether the study needed formal approval. Given the Dutch Medical Research Involving Human Subjects Act, the Ethics Committee decided that extensive formal approval was not needed for this study.

#### Co-researchers

At the start of the study, a recruitment letter and poster was spread to search for potential co-researchers willing to join the team. All potential co-researchers were informed in an individual meeting about the study prior to their decision to join the research team. They were initially informed about the purpose and procedures, the work, the voluntary nature of participation as a co-researcher, and the option of withdrawing at any time. Co-researchers then decided whether they really wanted to join and filled in a form with individual details needed for reimbursement and contact details. To ensure a meaningful participation of co-researchers, assistance was provided in several forms and ground rules for cooperation and confidentiality were drawn up together by the research team. All three research teams had discussions about confidentiality regarding individual client information and made agreements about privacy issues and collaboration issues that are described in the results section of this article. All co-researchers were also willing to contribute to the evaluation of the collaboration process.

#### Service users

Service users were informed by letter and verbally about the purpose for which the data collected would be used, the privacy agreements made by the research team, and the fact that they could withdraw at any time. Service users who were interviewed by co-researchers according to one of the five qualitative instruments participated after completing a consent form and agreed that an audio recording would be made. The consent form was also signed by the co-researcher who was performing the interview and by the researcher present for the observations. In the interviews with service users, a ‘process consent’ approach was adopted, meaning that we constantly observed whether consent was still present by paying attention to verbal and nonverbal indications of reluctance or hesitation to participate [42].

## Results

### Main overview

The following results section is divided into three sections. The first specifies the co-researchers’ motivations for participating. The second describes the main requirements for a good working environment and collaboration in participatory research. The third section gives a description of the differences within and between the teams. The characteristics of members of the three research teams are described in Table 2.

**Table 2:** Description of the three research teams

	Characteristic	Older adults team - OA	Mental health team - MH	Intellectual disability team - ID
**Co-researchers**	**Male / Female**	2 / 3	3 / 2	4 / 2
	**Living situation** **In- / outpatient care setting**	Situated in rural area 5 / 0	Living in Amsterdam (capital of the Netherlands) 0 / 5	Situated in and around a city in the province of Noord-Brabant 3 / 3
	**In care for**	5 physically frail elderly	2 people for addictions, 1 people for personality disorders, and 2 people with autism	5 people with mild intellectual disability, 1 person suffering from a non-congenital brain injury
	**Age** (years)	73–93	32–67	24–67
**Researchers**	**Male / Female**	1 / 1	1 / 1	0 / 2
	**Age** (years)	27 and 67	27 and 67	27 and 41
Five co-researchers and two researchers took part in the older adult research team (OA team). Co-researchers all lived in the same residential care facility in a small village. One co-researcher was a critical thinker and offered many ideas, while others were more accommodating and looked for a feeling of togetherness. The atmosphere was friendly, relaxed and low-paced. All co-researchers received some kind of support such as support with dressing and showering, cleaning, meals or medication provision. They were all able to move independently within the building. Co-researchers talked a lot about their experiences with the care provided and their lives before they entered the residential care facility.				
The mental health team (MH team) comprised five co-researchers and two researchers. The atmosphere in the MH team was generally very energetic. Co-researchers were very willing to contribute and think along; they had a lot of ideas and criticisms. The co-researchers could reflect very well on the research process and expressed themselves clearly. Three of the co-researchers received outpatient support, the other two co-researchers were in a stage of their recovery process in which they no longer received care. Three co-researchers used their experiences to assist service users with mental health issues in a paid position. Co-researchers had experience with a variety of psychological issues, among others autism, addiction, and personality disorders.				
The intellectual disability team (ID team) consisted of six co-researchers and two researchers. The atmosphere in this research team was generally very cheerful. The co-researchers were eager to learn and often asked questions. Some of the co-researchers reflected on the research process actively, whereas others preferred to listen to the ideas of other co-researchers. Most of the co-researchers were open in their communication, including about what they did not like when they provide feedback. Three of the co-researchers lived in a care facility themselves and three received outpatient support at home. Five of the co-researchers were born with their intellectual disability, and one co-researcher suffered from a non-congenital brain injury.				
In total, three researchers were involved. A young female PhD student (AS) with an educational background in interdisciplinary social sciences was part of all three research teams throughout the project. One researcher is almost retired and works for a Dutch client council organisation with a nationwide scope. He was first part of the ID team only and later on part of the MH and OA team. The third researcher (NB) is a female senior researcher of 41 years old having an educational background in public health and movement sciences. She was first part of the OA team and later on part of the ID team. The researchers were eager to perform the participatory research and put into practice their theoretical knowledge based on the literature. During the facilitation of the team meetings, researchers took on slightly different roles based on the features of co-researchers of each team. In the MH team, researchers ensured that all co-researchers could have equal contributions, and facilitated the process of seeking consensus in the team. In the OA team, researchers tried to stimulate co-researchers to think in a critical manner. In the ID team, researchers tried to hold on to a clear structure in the meetings to calm down the atmosphere.				

## Co-researchers’ motivations for participation

Co-researchers gave five motivations for participation that they found to have significant importance: being committed to quality improvement, being of worth to other service users, being part of a social group, creating a new social identity, and personal development / acquiring new skills.

### Commitment to quality improvement

Co-researchers shared substantive reasons for joining participatory research. One co-researcher described the role as a co-researcher as an essential bridge that connected individual experiences of service users to the level of a care organisation. In this way, this co-researcher was meaningful for other service users:

*“I thought it was really useful that you could be an important link between the stories and the complaints of the people and the organisations. That you’re able to do something for people.” (co-researcher 15, OA)*

Other co-researchers specifically underlined the desire to improve the quality of care relationships between service users and care professionals in long-term care, which was the main aim of using the qualitative instruments:

*“To try and help bring a client and professional together. I'm also intrigued to see if the relationship between the clients I interviewed and their supervisors are now better.” (co-researcher 6, ID)*

A couple of co-researchers told they were highly motivated right from the start to study the differences between the qualitative instruments that were evaluated in the study.

### Being of worth to other service users

For most co-researchers, it is important that they are valuable for other service users. Sometimes co-researchers heard stories of other service users who experienced problems in their relationships with care professionals. Co-researchers were motivated to help service users to improve the situation:

*“I think it's really important to be able to mean something to someone else. That you're living here and can genuinely change things for someone else.” (co-researcher 12, OA)*

Aiming to improve the quality of care was not only important to service users, but the co-researchers themselves also benefited because they felt meaningful and valuable for other service users, as well as for the community at large:

“*It’s nice to do something like that, in the final stage of my life. To be able to do something useful. I always used to make efforts to help people in my work. And now I still can. I hope we all get something out of it. Me, the residents, staff... the whole lot.” (co-researcher 12, OA)*

### Being part of a group

For some co-researchers of the three research teams, the social connections and social interactions with the research team members and social contacts with respondents were the primary reason for joining.

*“The group was a very positive experience for me. It gave me a lot of energy for being with the group.*” (co-researcher 2, MH)

Multiple co-researchers mentioned the nice and cosy atmosphere in the team, and the added value of the team support for overcoming their feelings of insecurity with their interviewing techniques.

### Creating a new social identity

Joining the research team presented possibilities for creating new social roles and acquiring a new social identity. For example, one co-researcher shared the experience of telling his family at a birthday party about his role as a co-researcher. The family members responded positively to the news. This reaction mattered a lot to this co-researcher. Other co-researchers were glad to join something so different from their usual day activities, and visiting places they had never been to before. Positive reactions from other people such as care professionals, service users, or friends boosted their self-esteem:

*“It's nice that people come to me to ask how this interview went. You can really see me improving!”* (co-researcher 6, ID)

### Personal development and acquiring new skills

Another theme that came to the fore in discussions within the three research teams was acquiring new skills. Being part of the research team created a safe environment for learning new things and generating self-esteem. For example, some co-researchers carried out tasks which they had not thought they could. Co-researchers also referred to the training and interviewing skills that they learned as useful assets:

“*I learned to keep asking questions. I mustn't impose opinions on others. I’ve stopped doing that.”* (co-researcher 8, ID)

Being involved in research changed the perspectives and ideas of co-researchers about some topics. Co-researchers described examples in which they observed their environment more critically after joining the research team, in order to see what could be improved. Moreover, participation made co-researchers reflect on their own activities and work

*“It makes me more aware. What am I actually doing at the moment? And my own position to come. I can now make a decision about what I want to do in the future.”* (co-researcher 1, MH)

Co-researchers also described how the research boosted their self-esteem:

*“The aim was to get more self-confidence and stand up for myself better. I can do that now. I'm more self-assured. Life has become more interesting. I know a lot more now than I did at first.”* (Co-researcher 3, MH)

In particular in the MH team, co-researchers saw their participation as an opportunity to gain more work experience. Several co-researchers requested a job reference that they could use when applying for jobs.

## Requirements for participatory research

The requirements for collaboration in participatory research were broken down into chronological order: for developing a good working environment and for the actual collaboration. These requirements were based on the perspectives of both researchers and co-researchers.

### Requirements for developing a good working environment

One of the lessons learned from the collaboration was the importance of developing a good working environment. Five requirements were found to contribute to a good working environment, which are outlined below.

#### Discussing reasons for engagement and wishes

Individual introductory meetings were held with service users who had shown an interest in joining the research team. Co-researchers then decided whether they really wanted to join. An introductory meeting was then organised in which all the members of each research team shared their reasons for becoming involved in the research (see previous section 1. Motivations) and shared personal information about their lives such as hobbies and interests. The research project and desired roles were also discussed. Discussing the reasons for involvement and sharing their wishes concerning the collaboration created a shared understanding of what each team member wanted to accomplish in performing the research together.

#### Formulating ground rules

Ground rules were decided on jointly in each research team and written on a flip-over. Agreements were made about privacy issues, team cooperation, and the possibility to stop taking part. This resulted in four agreements:

1. “*We do not talk about the shared experiences of clients in the research team. If it has no added value, we do not mention the names of clients in the research team*.”
2. *“Listen to the client: stop the interview when a client is too tired*.”
3. *“If you notice anything, tell the group. Or otherwise share it with the person involved or (researcher’s name).”*
4. *“If you are sick or too busy with other things, sign out. You can also stop (or stop temporarily).”*

We decided it would always be possible for a co-researcher to resign for personal reasons. Beforehand, the researchers expected that there was a chance that this would happen for these client groups, due to their long-term illness. In practice, the co-researchers were very often present, as they valued their participation highly. All team members tried to stay attentive about the first privacy agreement. Co-researchers also reported at the end of the research they found it really helpful “*to be honest to each other, and give your opinion*.” (Co-researcher 7, ID).

#### Training sessions

A training course of at least five sessions was given to each research team to prepare co-researchers for their active involvement in applying the qualitative instruments and evaluation. The specific content of the training sessions has been added as Appendix in Table 4. The training was prepared by the researcher (AS) and partly by one trainer who was hired for training interview techniques, based on the planned research activities, lessons of previous participatory studies, and wishes of co-researchers. The meeting length was adapted to the concentration span of the co-researchers, lasting between 90 and 120 min. Lay language was used as much as possible. Written information for the ID and OA teams was in a large font, in short sentences and simple language, and with more white space between the lines. If the co-researchers said they needed extra practice on some topics from the training, an extra meeting was planned. In all the teams, more time was taken to practice interviewing and using the qualitative instruments than scheduled. All team members completed the training. Afterwards, co-researchers said that the training had been really helpful in acquiring and developing the skills needed for interviewing. One co-researcher explained, “*The training has helped me understand interviewing, and I also learned how to apply the knowledge. I sometimes tended to fill in a question for someone. I’ve learned not to do that, and not to draw premature conclusions by asking questions ending with ‘right?’ or ‘isn’t it?’*” (Co-researcher 11, ID).

#### Availability researcher and travel support

Throughout the research project, one researcher (AS) could be reached by telephone, e-mail, and text messages. After a while, the researcher became familiar with the various needs of individual co-researchers as well. At the start, the researcher (AS) reminded all co-researchers of scheduled meetings shortly beforehand. After a while, some co-researchers still needed this extra reminder whereas others were perfectly able to remember meeting dates. If necessary, a researcher helped co-researchers organise travel to all gatherings and interviews. For example, some co-researchers asked whether their care professional could also be told about the meetings so that these care professionals could arrange transport by a tax or bus or could reschedule daily activities. For another co-researcher, the researcher pre-planned the route and sent the co-researcher a link to the online routing map to help the co-researcher to cycle from his house to the meeting location*.* This communication helped the co-researcher “*get a picture of what to expect and make clear where I would be going.”* (Co-researcher 4, MH).

#### Financial appreciation

A financial budget was available for paying co-researchers an allowance for their participation, but such an allowance was tied to national restrictions. As most co-researchers are either deemed unfit for the labour market or receiving a pension, they are receiving monthly payments from the Dutch government with a restricted maximum allowance for other activities. These co-researchers are only allowed to receive 1500 euros per year for their volunteering work, otherwise the reimbursement will be deducted from their benefit resulting in extra bureaucracy. In addition, travel expenses normally require a receipt for the tax reimbursement. In some cases, arrangements were made to reduce this administrative burden.

### Requirements for collaboration

Besides creating a positive working environment, good collaboration between team members was felt to be essential. Six requirements were identified that influenced actual collaboration in the teams. Each requirement is explained below.

#### Development of a bond

Developing a close bond between team members - including the co-researchers and researchers involved - was very valuable for good collaboration. This required spending time together in training sessions and work meetings and constantly listening to each other. There was a gradual progression of opening up towards each other, showing our true selves, our ideas and starting to trust each other. Team members got to know the character, needs, and the strengths and pitfalls of every team member. This knowledge made fruitful collaboration possible as team members could better estimate what could be expected from each other and build enough trust to share ideas and give each other feedback.

#### Deciding on clear role division

Before the start of the study, the exact division of roles was left open. The role splits were discussed and agreed upon from the beginning and during the study by the teams. Co-researchers found it quite logical that all perspectives of respondents, care professionals and (co-)researchers needed to be taken into account. The preparations were done in partnership, with co-researchers and researchers both participating and deciding together. The co-researchers were in control when applying the qualitative instruments, while a researcher supported when necessary. Each co-researcher held at least 5 interviews. During the analysis co-researchers shared their experiences, gave advice and participated in the discussions at team meetings. The advices given by the co-researchers, experiences of respondents and experiences of care professionals were synthesised by researchers. Although each perspective placed its own emphasis on certain aspects, the findings of the various perspectives were mostly in accordance with each other and there were no major differences in judging the qualitative instruments on their usability. Thus, the researchers had a substantial role as they kept the overview of all evaluation material and made sure that all perspectives were taken into account. In the dissemination of the findings co-researchers were actively involved by sharing their experiences in several presentations, presenting the findings on the congress which was organised specifically on the research findings of this study, and in the production of videos for dissemination of the instruments in the toolbox.

The first author had the responsibility for the planning and communicating the training dates, work meetings and the interviews. She was also responsible for the research planning and the progress and quality of the research. Although all team members were accountable for proper collaboration, the researcher also checked whether everyone agreed about the way the research was done together, or whether friction had arisen. Co-researchers and researchers improved their skills by performing the research together and the tasks of each team member changed accordingly as the collaboration matured. For example, one co-researcher said, “*At first I found it difficult to feel calm, and that made it difficult to keep my concentration during an interview. Now I can do this well, I reckon.”* (Co-researcher 1, MH).

#### Transparency and feedback

A third facilitator for collaboration was clarity and openness of transparency about the preferred and possible degrees of participation on either side. Points of friction could only be recognised and discussed when both co-researchers and researchers talked about the difficulties. Feedback on what was noticed during the interviews and about each team member’s role, and discussing critical or delicate moments and reflecting on them together, were essential for good collaboration. The conversations took place directly after each interview and at the research teams’ work meetings to reflect on shared difficulties and achievements. Team members often provided useful solutions or suggestions to each other for handling specific situations. Open communication and joint reflection require self-knowledge and generated a learning and development process that improved our research skills. The following example shows more clearly how this openness was achieved. One co-researcher in the ID team had little share in one interview, and left the questioning mainly to the other interviewer who was assisting in the interview. The available researcher suggested after a while that the co-researcher could take the leaflet with the questions from his bag as a reminder of possible questions to ask. Afterwards, the co-researcher told the researcher he did not like the fact that the researcher gave this suggestion, as he consciously had chosen to do the interview without leaflet. Thanks to this openness, the researcher and co-researcher were able to agree that the researcher would not make such suggestions in future interviews anymore, and would stick to the observation role as agreed beforehand.

#### Need for structure

During the research, some co-researchers preferred more structure than was given in the beginning of the collaboration. Co-researchers needed a lot of flexibility for dealing with interview cancellations by respondents. The last-minute changes and the range of unexpected events were the main barrier to participation, stated the co-researchers in our research. The researcher involved in the planning (AS) learned that providing certainty, clear information and as many details as possible were very important. This issue was regularly discussed in work meetings. One co-researcher stated: “*It did stress me a bit at times, though that’s partly down to me. I find it awkward if things aren’t clear when the interviews are being planned.”* (Co-researcher 6, ID). The researcher also communicated to co-researchers that it was sometimes difficult to give information in time when communication with a respondent was slow, or when there was a high workload in a busy week. Another co-researcher wanted to receive more information about the interview setting and the background characteristics of a respondent prior to an interview. Other uncertainties that were mentioned were unexpected violent emotions of a respondent in an interview, the unknown number of respondents attending a group interview, unexpected twists in a group conversation, and the waiting time before a respondent was able to start the interview.

#### Equal positioning

Equal positioning was an important factor for our collaboration, i.e. letting every team member have an influence and a say in the way the research was performed. At the beginning of our cooperation, the researchers learned a lot about this aspect from one event in the MH team that caused friction when writing the invitation letter for respondents. Two co-researchers were quite critical of how the draft had been formulated and gave a lot of comments, but they did not make changes to the text themselves. One week later, the invitation as modified by the researcher did not meet the expectations of the co-researchers. In retrospect, the co-researchers explained that they were angered by a remark the researcher had made (“*I’ll make something out of it*”) because they had worked it out in the meeting together. In the end, the team decided to plan an extra meeting to work on the invitation once more by sitting behind a laptop and writing the text together. This example was a good lesson for the researchers about creating new practices to counteract the traditional power imbalance and about the influence of the formulation and the use of an inclusive vocabulary. The skills of a researcher for equal positioning were summed up by the co-researchers of the OA team later on in the collaboration process: a researcher needs to be willing to cooperate, to be able to listen well, to let someone finish talking and not interrupt too soon, to accept opinions from others, and to be good at deliberation.

#### Sufficient time

Time for collaboration was found to be an essential requirement of participatory research. Substantial time and effort is needed from all team members. For co-researchers, it was sometimes difficult to combine an interview with other planned activities: “*When an interview is held in the evening, I’m tired afterwards. So I take that into account on beforehand, by planning fewer activities in the days before and after. I need time to process it*.” (Co-researcher 8, ID). Specifically for the researcher who is in charge for the planning and coordination, substantial time was needed for planning the interviews with respondents, co-researchers and the extra supporting interviewer (as the activities and schedules of every individual needed to be taken into account). Communication with co-researchers and taking account of the individual situations of co-researchers also required substantial attention from the researcher coordinating the study.

## Features of inclusive collaboration: individual contributions and team differences

Valuing contributions and differences at both the individual level and the team level was essential for achieving an inclusive collaboration. The skills and unique contributions of co-researchers and researchers and the differences between the teams are described in this section. The contributions of co-researchers and team differences were mainly based on the experiences of researchers, as the first author cooperated in all three teams; this created the possibility of identifying personal skills and group differences. The contributions of researchers were mainly based on the experiences of co-researchers.

### Personal skills and unique contributions of co-researchers

The abilities and individual contributions of co-researchers differed a lot. In the OA team, one co-researcher was very critical of the quality of the care organisation and was very accessible when respondents wanted to share negative feedback. Another co-researcher was really up-to-date and knew exactly what was going on in the care organisation; she knew almost all the residents personally. One co-researcher put a lot of effort into inviting service users to a group meeting, for which it was hard to attract service users. The co-researcher “saved” the group meeting by making multiple individual invitation rounds of possible respondents in his wheelchair. Thanks to the efforts of this co-researcher, enough service users attended the group meeting in the end.

In the MH team, one co-researcher was a really quick reader and remembered the structure very easily. She therefore did not have to look closely at the written interview instructions. Another co-researcher already had experience with group conversations with service users, and she had exceptional interviewing skills. She therefore supported the ID team in their interviews by summarising, making notes and asking probing questions. Two co-researchers independently decided to rewrite and summarise the original group interview instructions in their own words before their first focus group meeting. Afterwards, they were able to moderate the group discussions in a natural and personal way.

In the ID team, one co-researcher had a calm presence that let respondents feel at ease, but he asked probing questions less often. Another co-researcher formulated many of her own questions, was really good at holding on to the interview structure, and at providing extra explanations or examples for a question when a client did not understand it.

At the same time, there were also co-researchers who did not have a particular competency that stood out. This was especially visible when a co-researcher with less interviewing competencies interviewed a very strong client who was a quick thinker and talker. While the personal competences of some co-researchers did not stand out in terms of the value for the research itself, the personal value of participation seemed as high to them as to the others. The opportunity to contribute was also very important to them. For example, the care professional of one co-researcher told the researcher that they had spoken with pride about their experiences as a co-researcher, and the added value of their contributions to other service users.

### Abilities and contributions of the researchers

According to the co-researchers, the key ability for researchers was being able to support co-researchers in fulfilling their tasks. Before applying an instrument, the researchers helped co-researchers prepare by reading the structure together, practising the introduction of the method together, and appraising the abilities of co-researchers. After each interview, the researchers and co-researchers took time to evaluate the interview together. Researchers always gave compliments and also shared one learning point the co-researcher could work on. The co-researchers felt it was important that the researchers were not too critical. Correspondingly, the researchers always tried to let co-researchers go home with a good feeling about their contribution. In this sense, the role of researchers could be described as coaching. Other abilities of researchers, mentioned by co-researchers, were: being easily accessible by telephone, having personal interest in co-researchers and willing to show their own personality in the collaboration.

### Team differences

The three research teams differed a lot. In the OA team, co-researchers often related the stories they heard to their own experiences and values. This was specifically the case when their views on the interview outcomes were asked. The co-researchers were less used to abstract thinking and tended to use more common ground with their own experiences. Moreover, the cooperation and observations gradually showed (in the case of the majority of the research team) that co-researchers did learn interviewing skills less quickly, such as asking open and non-directive questions. But even after several interviews, their interviewing skills remained closer to their level at the start of the study. In the MH team, co-researchers were better able to make the distinction between research findings and personal experiences, and were quick learners and very self-reflective. They were capable of being openly critical on several aspects, regardless of the opinions of other members of the research team, including the researchers. Co-researchers had a clear picture of participation possibilities, other than conducting the research itself. In the ID team, the ability to keep their own experiential knowledge aside was also apparent, and they were non-directive in their method of interviewing after the training. However, deeper reflections were difficult for some co-researchers, while other co-researchers could also reflect on their own role and the interview results. The ID co-researchers evaluated their roles very optimistically, without a critical attitude. These co-researchers had neither the desire nor the abilities to interview independently.

Given the team differences, different kinds of support had to be provided arranged for holding the interviews and focus groups. On the whole, the teams agreed that every co-researcher would do one interview a day. The only deviation from this general rule was when a co-researcher wanted to try two interviews consecutively. In the MH research team, co-researchers decided to perform two qualitative instruments without extra assistance. In the ID team, an experienced co-researcher or professional interviewer assisted the co-researchers in all interviews by asking probing questions, summarising and writing answers. The OA research team held all the interviews for one qualitative instrument in cooperation with care professionals, whereas the design of the second instrument seemed easier and the team decided that co-researchers could conduct these interviews without assistance. During the interviews for the second instrument, the team found out together that the design alone did not provide sufficient support for co-researchers. The researcher, who was present for observing the interviews, stepped away from the observation role by helping and supporting co-researchers several times in interviews. In the evaluation, interviewing assistance was subsequently added as a condition for the application of this instrument.

## Discussion

This article describes lessons learned in the process of working with co-researchers in long-term care by reflecting on the process of participation and collaboration in participatory research. We tried to create insight into what helped and hindered co-researchers in making significant contributions. The motivations of co-researchers ranged from individual goals – such as personal development, creating a new social identity and belonging to a social group – to more external goals, such as being valuable for other service users and increasing the quality of care. Our research derived eleven requirements for participatory research for developing a good working environment and achieving a good collaboration. Moreover, an inclusive collaboration requires valuing the individual contributions of co-researchers and adjustment to team differences.

Several recent studies provide insight into participatory research with co-researchers of one specific client-group [13, 14, 26]. The present study evaluated collaboration with co-researchers in various long-term care settings, in order to report on lessons learned for future research. Due to the great emphasis attached to client participation recently, care organisations are expected to involve service users increasingly in quality improvement. These care organisations can do so by starting with small local initiatives for client participation in long-term care with those service users who can be easily involved. Researchers and employees of care organisations can use the findings reported in this study to design and shape inclusive future research and quality improvement projects, if they are willing to start a participatory study themselves in long-term care.

From the perspective of the researchers, individual and team contributions were worthwhile for the quality of the study, as illustrated by the wide range of contributions made by co-researchers during the study. As was shown by the motivations of co-researchers, they attached great personal value to the collaboration, corresponding to the ontological argument for participatory research that service users have the democratic right to be involved. The reported motivations of co-researchers correspond well with findings of previous studies [43]. Once co-researchers learned interviewing skills and grew in their role, they were very much willing and able to perform a variety of tasks. The fact that no co-researchers quit the collaboration during the performance of the instruments showed the high motivation of co-researchers, as did the appraisal co-researchers assigned to their roles. Their continued involvement could also be interpreted as a sign that the coordination and collaboration were carried out in such a way that co-researchers felt respected and appreciated, and that team members were well able to resolve the points of friction encountered during the collaboration, such as the need for structure.

Taking account of the benefits and added value of client participation, there are also some major demands and substantial efforts required when considering starting a participatory study in long-term care. In line with earlier research [15, 28], this study shows that participatory research is time-consuming and labour-intensive. Although research skills can be learned and used by co-researchers, coordinating and assistance remain essential and is dynamic and rather complex. In order to prepare co-researchers properly, it is therefore worthwhile to discuss the flexibility needed for performing research on beforehand. By overcoming these complexities of participatory research could be valuable in improving quality of care using co-researchers who represent the client perspective.

Furthermore, in line with previous studies [7, 21, 44], this study shows the diversity of long-term care service users, both within and between the three research teams. Three cases in different long-term care settings were selected to strive for maximum variation to be able to look into variations within- and between teams. The roles and task divisions were set up together in the teams in order to include the various voices of co-researchers with different strengths. This not only let the most literate service users join the research teams, but also involved a wider variety of co-researchers. The consequence of this recruitment approach was that not all co-researchers had or needed to have a particular competency that stood out. The personal value of participating was felt to be as high for these co-researchers as for the others. A recommendation for future study is to weigh up the democratic right to become involved in research and the minimum competencies needed for a particular study prior to recruiting co-researchers for participation in the study. As Bindels (2014) points out, ongoing reflection and room for change is needed as there is no single perfect collaborative method that applies to all co-researchers. Neither should every person necessarily be involved as a co-researcher [14]. Different methods and levels of client participation can be useful for achieving person-centred care in long-term care, depending on the content and goals of a participatory study [45]. A related issue was that, similar to other participatory studies, co-researchers chose roles that were meaningful to them and mutually beneficial to the group, without pressure to participate in tasks that have little meaning to them such as academic writing [15]. The motivation and willingness of one co-researcher to be involved writing the academic article, is an example of how each co-researchers used their own competences and interests in their contribution.

In the current study, some co-researchers, especially of the MH team, had exceptional interviewing skills. These co-researchers started to assist co-researchers of the other teams with the interviews. The question could perhaps be raised of whether these highly competent co-researchers from the MH team could conduct all the interviews, including the interviews for the other client-groups. Although this would have been practically beneficial, our study indicated the opposite based on two observations. Firstly, respondents could recognise themselves better in a co-researcher with the same care needs. This central characteristic would be lost if a MH co-researcher performed all the interviews individually. Secondly, the co-researchers of the MH team repeatedly showed a lack of knowledge of the specific situations and contexts of the other client groups, sometimes resulting in a lack of empathy for a client situation or an awkward moment. Including co-researchers with diverse backgrounds as representatives of different client groups therefore remains fundamental: as interviewing skills alone are not enough.

### Methodological considerations

Some strengths and limitations can be identified in this study. A strength was that experiences of both co-researchers and researchers were included, as both were needed to achieve an effective and fruitful collaboration. From our perspective, it is not possible to provide a complete picture of our collaboration by focusing on the value and contribution of co-researchers alone, as one of the two parties will then not be described [7, 29]. Partnerships need to be reciprocal and all aspects of participatory research – from the initial training to the manner of reflecting together and reporting the findings – should contribute to this reciprocity [19]. In line with this, explicitly including both the co-researchers’ and researchers’ perspectives in this manuscript could be seen as a strength. At the same time, it means that the article was written based on our own reflections on the collaboration from an insider’s perspective. As is outlined in other research, this could be seen as both a strength and a limitation at the same time [3]. One limitation of the convenience sample in forming the teams is the limited possibility for making clear distinctions between the research teams that represent three client groups of long-term care within the teams. It is for instance not possible to draw conclusions on the differences between the motivations for collaboration between the three teams as if they were typical for the distinct client-groups. With regard to abilities, there were clear differences visible between the three research teams, but other co-researchers from other client groups may show other specific strengths and capacities in future research. This has implications for the limited generalisability of the findings related to client group differences. Another limitation was that the co-researchers and researchers were included after the research proposal had been submitted and approved. Although a lot of details were left open in the proposal (choice of qualitative instruments, content of qualitative instruments, distribution of roles), this created a power and information imbalance at the beginning of the research project, resulting in less decision-making space with regard to the main goals and focus of the research project [14].

Several topics for future work arise from this study. As the current study described the collaboration in three research teams, more good practices could further increase the accumulation of knowledge about the opportunities and barriers in participatory research focused on people with intellectual disabilities, older adults and people with mental health issues. This means it would be relevant to focus on developing research skills and contributions of co-researchers in long-term research projects, thus creating insights into the learning curves and the skills of long-term care client groups. For the implementation of instruments for participatory quality improvement initiatives in a Dutch context, a toolbox with training materials and a guideline for implementation is available in Dutch on the website www.nivel.nl/toolbox-hzs. Moreover, it would be interesting to study whether care professionals are open to this new acquired role in quality improvement, and whether they are willing to reflect on the findings brought to the fore by co-researchers and if they could respect their position as equals.

## Conclusion

This case study describes the lessons learned from the collaboration and participation of co-researchers and researchers in three research teams in long-term care. The results showed the importance of developing a good working environment and establishing a good collaboration for participatory research. Furthermore, the study shows that individual and team differences should be taken into account. These results can be used by researchers for designing and shaping future research projects in long-term care in collaboration with service users as co-researchers.

## Data Availability

The data analysed in the current study is available from the corresponding author on reasonable request.

## Appendix

**Table 3:** Summary of study findings and lessons learned from this participatory research

	Co-researcher level	Researcher level	Process level
***Facilitators***	- *Individual preparation:* reading the instructions, practising asking probing questions, repeating the introduction, reflecting on past interviews. - *Being motivated* for one or more of the following reasons: being committed to quality improvement, being valuable to other service users, being part of a social group, creating a new social identity, or personal development and acquiring new skills, wanting to spend time. - *Becoming experienced:* doing research activities more often makes it easier.	- *Supportive attitude:* support when necessary, increasing self-confidence of co-researchers, backup. - *Coordination and communication*: inform co-researchers on time about the day and time an interview is planned, know what to expect, preparation of team meetings by researcher. - *Motivated* for participatory research.	- *Discussing reasons for engagement and sharing wishes for collaboration*: this created a shared understanding of what each team member wanted to accomplish in the research. - *Formulation of ground rules:* i.e. privacy issues, team cooperation and the possibility of cancelling attendance. - *Training*: learning to ask probing questions, learning to listen openly, practise in a realistic setting, written information in a small booklet. - *Preparation right before the interview*: Preparing the interview together: what are we going to do? Practising the introduction to an interview. - *Debriefing right after the interview:* complimenting each other, giving suggestions, and sharing what went well, what could have gone better. Discussing the report notes of a co-researcher. - *Collaboration in team:* being part of a team, joint meetings, good atmosphere, development of a bond, transparency and providing feedback, support of the team, sociability, good communication.
***Barriers***	- *Limited energy:* interviews sometimes took a lot of energy, and a lot of stuff to think about. Schedule extra rest moments and plan one interview for one day. - *Being a perfectionist*: set too high goals for yourself, becoming too nervous. - *Writing a report afterwards*: not motivated enough to write a report of the interview directly afterwards. - *Poor health and bad hearing:* (specifically in the OA team) bad hearing or poor physical health.	- *Permanent availability* for questions and travel support when needed, sometimes it was not possible to answer the telephone during another interview. - *Coordination and collaboration takes substantial time*	- *Ambient soundings and stimuli* during an interview, for example when a respondent chose an interview setting outdoors. - *Clarity on preferences* versus *flexibility needed:* interviews took place at various times which needed a lot of flexibility from co-researchers, whereas they preferred clarity beforehand, as early as possible. Some co-researchers preferred more structure..
***Requirements for participation***	- *Knowing service users of the care residence*: this makes inviting respondents easier. - *Motivation and skills*: Co-researchers need to be motivated for collaboration and quality improvement, have people knowledge, be social,good communication and listening skills, be enthusiastic, be interested in others, be healthy, able to hear, open to others. - *Courage:* Be brave, willing to overcome limitations. - *Relax*: try to stay calm and “remember to keep breathing in and out”.	- *Motivation and skills for equal collaboration:* willing to work with co-researchers, able to listen well, let the co-researcher have their say without interrupting, accepting opposite opinions of co-researchers, good in deliberation and open attitude in discussing themes.	- *Proper training:* practising together, getting to know the subject and each other. - *Coordination:* one person needs to be responsible for planning the interviews, for logistics, and for keeping an overview on the activities. - *Financial appreciation* for the time invested by co-researchers and a travel allowance. Pay attention to national restrictions for co-researchers receiving a pension or welfare benefit. - *Support* in the care organisation at the management level for the quality improvement initiative. Help in recruiting respondents and informing care professionals. - *Good collaboration:* team members fit in the team

**Table 4:** Contents of each training session

Training session	Content of the session
1 (introductory meeting)	Team members introduce themselves by using association cards
	Discussing the content, aims and need for the research (i.e. added value of client participation and evaluating the quality of a care relationship)
	Sharing motivations for becoming involved
	Formulating ground rules for cooperation
	Exchanging personal information for getting in contact and paying remunerations
2	Discussing research activities and role preferences Drawing up the invitation letter for respondents
	Modifying the content of the qualitative instruments: open or semi-structured questions
3	Training in individual interviewing techniques
	- asking open questions and multiple choice questions
	- techniques for encouraging a respondent to tell more
	- summarising
	Learning to present the research to respondents
	Completing an interview
4	Training in group interview techniques (optional)
	- asking open questions and multiple choice questions
	- learning to deal with group dynamics
	Practising observing nonverbal information such as emotions
5	Specific training sessions in applying one qualitative instrument, concerning individual interview and/or group interview techniques.

## Abbreviations

OA team: Team consisting of physically or mentally frail older adults
MH team: Team consisting of people with mental health problems
ID team: Team consisting of people with an intellectual disability
